# Giving birth in Switzerland: a qualitative study exploring migrant women’s experiences during pregnancy and childbirth in Geneva and Zurich using focus groups

**DOI:** 10.1186/s12978-019-0771-0

**Published:** 2019-07-22

**Authors:** J. Sami, K. C. Quack Lötscher, I. Eperon, L. Gonik, B. Martinez de Tejada, M. Epiney, N. C. Schmidt

**Affiliations:** 1 Faculty of Medicine, University of Geneva, Geneva, Switzerland; 2 Clinic of Obstetrics, University Hospital Zurich, Zurich, Switzerland; 3 Obstetrics Unit Department of Obstetrics and Gynecology, Geneva University Hospitals, Geneva, Switzerland

**Keywords:** Maternal health, Pregnancy, Migrant woman, Qualitative study, Focus group

## Abstract

**Background:**

Migrant mothers in high-income countries often encounter more complications during pregnancy, delivery, and the postpartum period. To enlighten health care providers concerning potential barriers, the objective of this study was to explore positive and negative experiences with maternal health services in the University Hospitals of Geneva and Zurich and to describe barriers to maternity services from a qualitative perspective.

**Methods:**

In this qualitative study, six focus groups (FGs) were conducted involving 33 women aged 21 to 40 years. All FG discussions were audio-recorded and later transcribed. Data were analysed using a thematic analysis approach assisted by the Atlas.ti qualitative data management software.

**Results:**

Positive experiences included not only the availability of maternity services, especially during emergency situations and the postpartum period, but also the availability of specific maternity services for undocumented migrants in Geneva.

Negative experiences were classified into either personal or structural barriers. On the personal level, the main barriers were a lack of social support and a lack of health literacy, whereas the main themes on the structural level were language barriers and a lack of information.

**Conclusion:**

Structural adaptation is necessary to meet the needs of the extremely diverse population. The needs include (1) the provision of specific information for migrant women in multiple languages, (2) the availability of trained interpreters who are easily accessible to health care providers, (3) specifically trained nurses or social assistance providers to guide migrants through the health system, and (4) a cultural competence-training programme for health care providers.

## Plain English summary

Migrant mothers in high-income countries often encounter more complications during pregnancy, delivery, and the postpartum period. The objective of this study was to explore positive and negative experiences with maternal health services in the University Hospitals of Geneva and Zurich and to describe barriers to maternity services. The study used a qualitative approach, involving 33 women aged 21 to 40 years in six focus groups. The data was analyzed, and common themes were identified.

Positive experiences included the availability of maternity services, especially during emergency situations, but also the availability of specific maternity services for undocumented migrants in Geneva.

Negative experiences were a lack of social support, a lack of health literacy, but also language barriers and a lack of information.

In conclusion, the study suggested the following four structural adaptations:

1. the provision of specific information for migrant women in multiple languages
2. the availability of trained interpreters who are easily accessible to health care providers
3. specifically trained nurses or social assistance providers to guide migrants through the health system
4. a cultural competence-training programme for health care providers

## Introduction

In 2017, 258 million individuals worldwide were living in a country other than their birth country [1]. The International Organization of Migration (IOM) defines a migrant as “a person who who moves away from his or her place of usual residence, whether within a country or across an international border, temporarily or permanently, and for a variety of reasons”, regardless of the person’s legal status and whether the movement is voluntary or involuntary [2].

Almost one-third of the world’s international migrants (78 million) lived in Europe in 2017 [1]. The same year, the canton of Geneva and the city of Zurich reported that 40.2 and 32.4% of their respective residents had a nationality other than Swiss, and these values were higher than the Swiss average of 25% [3–6].

Adapting to a new country can raise important challenges, and the previous literature has described health disparities between migrants and host populations [7, 8]. Among other issues, migrants have been reported to be more susceptible to suffering from diabetes, mental illness and maternal and child health problems [9].

Furthermore, studies have found that migrant women encounter more complications during pregnancy, childbirth and the postpartum period [10, 11]. In Switzerland, these observations have been confirmed by quantitative studies that have shown a higher maternal mortality rate among non-Swiss than among Swiss women and higher rates of unwanted pregnancies and delayed prenatal care in undocumented women [12–14].

Factors leading to these health disparities are difficult to disentangle, and multiple reasons have been proposed, including language barriers, a lack of health literacy and a lack of information [15]. A Swiss qualitative study by Bollini and colleagues explored maternity experiences among Turkish and Portuguese women in Switzerland and reported that these groups faced stressful situations that negatively affected their pregnancies [16]. Furthermore, a poorer level of satisfaction with maternity services may be a barrier for utilization of these services by pregnant migrants [15, 16].

To reduce maternal health disparities, their causes must be understood. Therefore, a qualitative study design was chosen with the aim of describing the experiences of migrant women with pregnancy and maternity services at two main hospitals of Geneva and Zurich and to identify specific barriers for health care accessibility.

## Methodology

### Study design and sampling

Between September 2015 and February 2016, a qualitative study using focus groups was conducted with migrant women who were either pregnant or in their first year after childbirth in Geneva and Zurich. Focus group are a common method in qualitative health research to capture insights related to the ways people perceive and interpret their surroundings [17, 18]. Each focus group (FG) was led by two researchers. While one researcher facilitated the discussion based on a semi-structured questionnaire, the other observed group dynamics and body language. This approach has been chosen as an adequate method because it reduces for example the influence of the researcher [19].

Discussion topics included 1) health during pregnancy, 2) experience with the Swiss health care system during pregnancy and childbirth, 3) perceived barriers, and 4) suggestions for improvement of the current situation.

Multiple recruitment strategies to access women during pregnancy or in their first year postpartum were used, including personal contact during antenatal care visits, contacting migrant organizations by email or telephone and the snowball method.

Six focus groups (FGs) were conducted with a total of 33 women, who had moved to Switzerland. Migrant women included in the study moved away from their home countries to one or more of the following reasons:

- Woman had left her country involuntarily either due to war, conflict or economic pressure.
- Woman lived in Switzerland without a legal status.
- Woman had recently arrived.

For women having migrated for one or more of the mentioned reasons, difficulties in accessing health services in Switzerland have been described in the literature [12, 14].

Additional characteristics of the FGs and participants are noted in Tables 1 and 2.

**Table 1:** Focus group characteristics

FG	Number of participants (total = 33)	Number of women (pregnant or postpartum)	Country/Countries of origin	Language of the interview (assisted by an interpreter)	Location
1	2	2 postpartum	Portugal	Portuguese (interpreter)	Zurich
2	4	2 pregnant	Peru, USA, Germany	English	Geneva
		2 postpartum			
3	5	2 pregnant	Dominican Republic, Peru, Bolivia	Spanish	Geneva
		3 postpartum			
4	6	6 pregnant	Brazil	Portuguese (interpreter)	Geneva
5	4	2 pregnant	Eritrea	Tigrinya (interpreter)	Zurich
		2 postpartum			
6	12	7 pregnant	Bangladesh	Bengali (interpreter)	Zurich
		5 postpartum

**Table 2:** Participant characteristics

	*n* = 33 (%)
Women 21–40 years of age	33 (100%)
Mean age in years (SD)	29.2 (5.5)
Mean years in Switzerland	
-less than 4 years	18 (54.5%)
-more than 4 years	15 (45.5%)
Religion	
Christian	15 (45.5%)
Muslim	11 (33.3%)
Other	2 (6.0%)
No answer	5 (15.2%)
Civil status	
Single	2 (6.1%)
Married or in partnership	29 (87.9%)
Separated or divorced	1 (3.0%)
No answer	1 (3.0%)
Education	
Less than 12 years	3 (9.1%)
High school (=12 years)	18 (54.5%)
Education > 12 school years	10 (30.3%)
No answer	2 (6.1%)
Children	
Expecting first child	7 (21.2%)
Already had children	26 (78.8%)
Work situation	
Full time or part time	10 (30.3%)
Not working	20 (60.6%)
Other (student or inability to work)	2 (6.1%)
No answer	1 (3.0%)
Health insurance	
Yes	17 (51.5%)
No	10 (30.3%)
No answer	6 (18.2%)

### Data collection and analysis

The study was conducted in the Services of Obstetrics of the University Hospitals of Geneva and the Department of Obstetrics of the University Hospital Zurich. Geneva and Zurich are two of the most important cities for immigration in Switzerland, and therefore the experiences of migrant women during pregnancy are considered of high importance for health care providers [5, 6]. The Ethical Commissions in Geneva and Zurich approved the study.

The FGs were led by one of the main investigators (NCS or KQL). After obtaining written consent from each participant, the FGs were conducted in private rooms in the University hospitals in either English or the maternal language of the participants with the assistance of a skilled interpreter who was accepted by the group.

The FG discussions were recorded, transcribed and translated into English. The transcripts were analysed using ATLAS.ti. CAQDAS and coded by two different members of the research team independently to avoid subjectivity. After individual coding, both researchers identified the main topics and sub-topics common to every group and outlined the women’s experiences with the Swiss health care system as well as the identified barriers.

Our study adopted the conceptual framework of Higginbottom and colleagues [20]. In this study, published in 2016, experiences of immigrant women with maternity care services in rural and urban Alberta, Canada, were investigated. Barriers were structured at the patient (personal) and maternity services (organizational) levels. The conceptual framework of Higginbottom et al. was chosen because identifying barriers especially on the organizational level enables health care institutions to make adjustments in order to provide equitable healthcare services for an increasingly diverse population [20].

## Results

The 33 participants were all born outside of Switzerland and had been in Geneva or Zurich for an average of 5.5 years. They originated from nine different countries, were on average 29 years old (range 21 to 40 years) and most were married (89%). Their education level was good, with more than 80% having completed high school. Nine out of 15 participants in Geneva and one participant in Zurich were living in Switzerland without a legal status and didn’t have a health insurance (Table 2).

The results are organized into two parts. In the first part, migrant women’s good experiences during maternity care are outlined. In the second part, the six major themes influencing the participant’s access to maternity care services are described and classified into either barrier on the patients or maternity service level.

### Good maternity care experiences

#### Pregnancy check-ups and availability of services in case of perceived emergency situations

The participants appreciated the availability of health care services and the possibility of calling health care providers (HCPs) with questions or perceived emergency situations. H. from Eritrea stated: *“I would say it’s better in Switzerland, because no matter what time it is, when you feel sick, you can call a doctor and you get an appointment for the next day right away.”*

Furthermore, most participants felt reassured by the number of pregnancy check-ups in comparison to those in their home country. S., who was 6 months pregnant and had given birth to two children in Brazil, said*: “I think here is better than there, because here they really make efforts to know how we feel, how we are, etc. In Brazil, I had an ultrasound during the first month of my pregnancy, and then at the end of it, when I was about to give birth. On the other hand, here I am six months pregnant and this is already the fifth ultrasound, because they want to be sure about the baby, how is the baby, if everything is alright …*”.

#### Postpartum period

In Switzerland in 2015 and 2016, all women with health care insurance, which is mandatory for legal residents, were entitled to a daily visit with a midwife until the tenth day after birth, even when at home. The midwife helps to care for the women, provides support with breastfeeding and answers their questions.

Most participants who had previously given birth in Switzerland appreciated the postpartum care services provided by the midwives. S. from Brazil stated: “*Once you give birth, a midwife comes. In Brazil, it is different. When I had my baby, I was 16 years old and I had to do everything by myself.”*

#### Specific services for women without legal status

Nine out of 15 women in the FGs in Geneva were without legal status. The women without legal status appreciated a service available only at the Geneva site that helped undocumented migrants’ access medical care. It also facilitated access for undocumented pregnant women to the University Hospital, where a specific antenatal care consultation was provided.

### BARRIERS on the patient side or the maternity service level

#### Patient side

##### Lack of social support from family members

In nearly all of the FGs, the women described a lack of social support and a feeling of loneliness. S.B. from Eritrea explained how she felt: *“When you have your parents with you, they are there to help because they feel responsible for you. Therefore, I find it harder in Switzerland compared to Eritrea.”*

##### Lack of health literacy

Health literacy has been defined by the World Health Organization as « the cognitive and social skills which determine the motivation and ability of individuals to gain access to, understand and use information in ways which promote and maintain good health » [21]. Women coming from countries with less developed health systems and lower education sometimes misinterpreted medical procedures due to a lack of information, but also the ability to understand the information. They were worried about the consequences of medical examinations. S.B. from Eritrea said: “*Yes! I mean maybe it’s not intentional. It could be that when the doctor puts instruments inside of you to check the baby, he or she accidentally damages the foetus, which in turn may lead to a miscarriage …*”.

Furthermore, during several FGs, divergent perceptions and misunderstandings with respect to the first trimester screening were described. The first trimester screening test, which is routinely offered in Switzerland to all women between 11 to 14 weeks of pregnancy to assess the risk for trisomy 13, 18 and 21 in the unborn, includes a blood test combined with an ultrasound. However, misunderstanding of these procedures led to anxiety and in some cases to refusal. V., a Brazilian mother, explained the reason for her refusal: *“Yes, but she* [the doctor] *didn’t explain to me right how this exam would be, and I thought it was the one with the needle, that’s why I said no and asked her if I could do it later.”*

The calculation of risk was also difficult to understand for several participants. As C. from Germany explained: *“I said yes, for me if the child is definitely sick I want to know and if he’s definitely not sick I want to know, but everything in between, I don’t want to know, I’d rather just have it and deal with it afterwards.”*

Other women reported not wanting to undergo this test because the result would not change anything, but they felt pressured to stop their pregnancy if the risk for a medical pathology was elevated. C.E. from Brazil said: *“Here, when you say I am pregnant, they say: here* [in Switzerland], *you can abort. It’s some sort of pressure for us. That’s what I find different. For example, in Brazil, for our culture and our people, if God sends us a baby that has some problems, we will still have the baby. Here, seems that there is a pressure if the baby has problems, you can just remove it.”*

##### Perceived discrimination

During the FGs, several women described a feeling of discrimination, especially if they were living in vulnerable situations in Switzerland, such as difficult social or financial conditions, or if they were living without a legal status. L.S. from Brazil felt pressured by the social worker at the hospital to stop her pregnancy: *“No, I think they do that because we are not married. In my case, I felt it happened because I was alone, because they suggested it several times... I think it’s because of a financial matter.”*

Additionally, a feeling of discrimination was reported during pregnancy check-ups. In University Hospitals, where most of the migrant women went for their maternity care, medical students can attend to consultations with the patient’s permission. Several women from different FGs explained that they thought doctors allowed students to attend the consultation mainly because they were migrants.

#### Maternity services level

##### Language barrier and lack of information

The main barriers at the maternity service level were language barriers and lack of information. Not being able to express oneself properly because of language differences negatively influenced the doctor-patient relationship and led to frustration or worries. Participants expressed the need to have more information about pregnancy in Switzerland available either in English or in their native language, because they were not able to ask questions or did not understand the answers. K. from Peru explained: *“I don’t know how it is here, what to eat or not eat, what should I drink... if I’ll understand the doctor, if they’ll understand me, because I don’t speak French”.*

##### Administrative procedures

Migrant women described difficulties understanding the administrative procedures of the Swiss health care system. These difficulties were in some situations closely related to a lack of health literacy on the patient side or a lack of information. N. from Peru noted: *“I think the doctor assumes that you know everything. I didn’t know that maternity services were covered 100%. I found out when my first bill came. Probably, there is information, but I don’t know where to find it.”*

In addition, women described that scheduling medical appointments was sometimes difficult and time consuming, especially for pregnant women who did not speak the native language. One woman who resided in Geneva and did not speak French explained: *“But you got to find it (the information) and know it’s there and kind of access it. It takes a lot of effort to cross that threshold. It takes a lot of energy and that’s the other thing: you’re tired all the time! You have to call someone that you don’t know, call them in French and try to schedule an appointment.”*

##### Lack of continuity of care

For several participants, not being able to stay with the same HCP during the whole pregnancy was difficult. This situation is especially the case for women with basic health insurance, because they cannot give birth with their doctor if he does not work in a public hospital. N. from Peru explained: *“The only problem is that he (her gynaecologist) works only at a private clinic, not at the public hospital, so I already said goodbye to him about 2 weeks ago.”*

Furthermore, women expressed their concerns about the constant change of HCPs once they arrived at the public hospital, as R. from Bolivia explained: *“I think it’s never the same doctor from one ultrasound to the other. That is difficult.”*

## Discussion

The current study is, to our knowledge, among the first in Switzerland with the aim of understanding experiences with maternity care services among pregnant migrants in two major cities for Switzerland from a qualitative perspective. Two main themes emerged in respect to the positive experiences: the availability of services in perceived emergency services and the postpartum care services. Six main themes were identified when women described their negative experiences. Those were categorized either in barriers on the personal or structural (maternity level) side: lack of social support from family member, lack of health literacy, perceived or real discrimination, language barriers and lack of information, administrative procedures and lack of continuity of care.

In general, our results in respect to perceived barriers are fairly consistent with previous national and international literature. However, until now few studies have described positive experiences. The results of our study showed that migrant women highly appreciated the availability of maternity services, especially in perceived emergency situations, and the postpartum care offered by midwives at home. In particular, women who had previously given birth in countries where routine postpartum care did not exist highlighted this service.

Furthermore, undocumented women in Geneva were aware of and appreciated the dedicated services provided for them by specific trained nurses and social assistants at the maternity hospital. This service is especially important, because the previous literature has reported that undocumented pregnant women have poorer access to pregnancy screening and check-ups [12, 22]. A recent paper by Balaam et al. showed that caring relationships (including those in health care) might help migrant women access maternity care [23]. Therefore, the availability of services such as in Geneva that are adapted to women’s needs may allow equity in accessing maternity care for migrants, including undocumented patients.

However, besides the positive experiences, several negative experiences and barriers were described. One important barrier mentioned by nearly all participants was the lack of social support. Most migrants explained that in their home countries women were supported during pregnancy, childbirth and in the first weeks after childbirth by their parents or family members. As parents or family members remained mostly in the home country, women described anxiety and loneliness. These findings are in contrast to a previous study conducted in Geneva among migrant women who did not mention a lack of social support [24]. One possible explanation might be the length of residency in the recipient country. Over 50% of the women in the current study had been in Switzerland for less than 4 years, whereas in the previous study over 60% of the participants had lived in Geneva for more than 4 years. Length of stay in a new country has been mentioned has an important factor for acculturation that allows among others the understanding of a new health systems [25]. Furthermore, this study included women during pregnancy or in the first year after childbirth. Family members and close friends might be perceived as especially important during this period.

Lastly, the type of migration experience needs to be considered. Several women had left their home country due to war or conflict. Women who have experienced war or even sexual violence are more at risk to suffer from physical or mental health problems [26]. In cases of involuntary migration, women might miss the social support from their family members even more. The same holds true for undocumented migrants, who participated in the study and for whom family unification was not possible. Health care professionals should be aware of this barrier, because a lack of social support has been linked to a higher risk of postpartum depression [20, 23, 27–29]. Even though the assessment of postpartum depression was not part of our study, evaluation by health professionals and the provision of support becomes especially important [23, 27, 28].

A second important barrier on the patient side was the lack of health literacy. “Health literacy” has been defined by the World Health Organization as “the cognitive and social skills which determine the motivation and ability of individuals to gain access to, understand and use information in ways which promote and maintain good health” [21]. Women often lacked information about the Swiss health care system, including administrative procedures, such as scheduling medical appointments. These observations align with findings from previous studies in maternity care [15, 20, 24]. Even if maternity services were equally available for all women, in practice the services were often not accessible to migrant women due to a lack of awareness about their existence [20]. Additionally, the institutional culture of maternity services has been reported to have been mainly designed for those who understand and can negotiate the system. Therefore, people who recently have arrived and are not familiar with the system face a range of barriers [30, 31].

Interestingly, none of the undocumented pregnant women in Geneva mentioned difficulties in scheduling appointments or the lack of continuity of care as a barrier. A possible explanation may be the dedicated services for them mentioned above, which guide undocumented migrant women through the system and provide antenatal and postpartum care. These observations were in accordance with those from a recent study in Switzerland, which assumed that those services might be helpful for migrants who had arrived recently and were not yet familiar with the system [24].

In our study, a lack of health literacy represented a source of worries for pregnant women, because they were sometimes not used to the medical procedures routinely offered in Switzerland. The consequent misunderstandings negatively influenced the patient-doctor relationship and were previously described as an important barrier to the use of health services [9, 15]. This finding is particularly important, since some HCPs are not aware of the lack of health literacy as a barrier and fail to explain medical procedures adequately to their patients. Therefore, increasing HCP awareness of women’s perceptions of health services through a cultural competence training programme in necessary, as has already been mentioned previously [30, 32]. Aside from the importance of setting up culturally sensitive care, the misunderstandings towards first trimester screening also show the importance of providing clear information to migrant women, as discussed further.

Furthermore, the lack of health literacy in our study was closely related to the lack of information, which might limit access to health care [33]. The inadequate provision of informative material was one of the main barriers at the organizational level. Guaranteeing “the same access to information to the native and migrant populations” has been reported by Bischoff as an essential intervention to promote health for migrant patients [30]. This finding is in accordance with our study and previous studies that have highlighted the need for migrant women to access information in their first language [31, 34]. Indeed, because recommendations during pregnancy may vary from one country to another, every woman should receive information resources for the host country provided by the HCP in her first or any familiar language [14].

The final barrier, which influences directly women’s health literacy, was suboptimal communication between HCPs and migrant women. This barrier is mainly a result of insufficient language proficiency on the patient side, which is necessary to understand the information provided by and to communicate successfully with the HCP. As highlighted by previous studies, language is one of the main barriers for health care access and satisfaction with on-going care as misunderstanding leads to lack of information and stressful situations [9, 15, 20]. Our study confirmed previous results showing that women relied mostly on ad hoc interpreters, such as family members, to overcome this barrier. This issue is especially important, because the use of ad hoc interpreters has been proven to lead to poor quality communication and a lack of confidentiality [33, 35, 36]. None of the participants in our study seemed to be aware of the provision for professional interpreters free of charge at the University Hospital Geneva and University Hospital Zurich. Therefore, efforts should be undertaken by health institutions to increase awareness of the availability of such services [37].

Furthermore, health care institutions need to address the barriers described on the structural (maternity) level and raise the awareness of health service providers in respect to those on the patient side. Therefore, the following strategies are suggested to reduce some of the most important barriers at the maternity level: (1) specific information should be provided for migrant women in multiple languages and actively delivered, (2) the availability of trained interpreters must be highlighted and made easily accessible to HCPs to overcome language barriers, (3) the use of professional social accompaniment, such as help with administrative procedures, should be considered, and (4) a cultural competence training programme for HCPs should be implemented with the aim of improving communication, increasing awareness of migrant women’s perceptions of health care and preventing discrimination.

### Limitations of the study

Although this qualitative study was among the first to explore barriers to maternity care services in the canton of Geneva and the city of Zurich, it faced several limitations.

First, the qualitative approach using focus groups covers the range of topics considered important by the participants but cannot be generalised. Also, the methodology of a focus group design might have prevented some participants to express their honest and personal opinion. However, to limit this influence, small groups with participants from the same social background were chosen. Furthermore, FGs were conducted in a private and confidential setting.

Second, migrant women present a very heterogenous group with different needs and experiences. Even if the study assessed for example the length of the stay in Switzerland and the legal status, future studies should include also information about the type of migration experience (forced vs. voluntary) or the length of residence in the recipient country [38]. Also, as already outlined, migrant women who have been forced to leave their home country, might have different expectations, experiences and health problems compared to those having migrated for economic opportunities. Additionally, the authors of this article do not belong to an ethnic minority group. Even though the authors tried to reduce those barriers by discussing them with representatives from migrant communities, an interviewer bias from a western perspective cannot be excluded.

Finally, understanding the experiences and perceived barriers of Swiss pregnant women or those in their first year postpartum would have been helpful to clarify whether organizational barriers are specific to migrant women or are also present for Swiss women.

## Conclusion

The Swiss health care system is meant to provide health care for everyone irrespective of their origin and legal status. Even if this goal holds true for maternity health services in theory, the barriers described in our study demonstrate that maternity health services are not always accessible. Although qualitative results cannot be generalized, we believe that our results are confirmed by the national and international literature. Therefore, reducing these barriers may be beneficial at personal and institutional levels and may support health system strategies to improve health equity. Barriers are strongly influenced by personal factors, but organizational barriers also exist. In our opinion, the latter are especially important, because policy makers and HCPs can act to overcome them. Strategies to reduce some of the most important barriers at the maternity level should focus on language barriers and the improvement of health literacy, but also address perceived discrimination and administrative procedures.

## Data Availability

The datasets (transcripts) generated and analyzed during the current study are not publicly available due to the sensitivity of the data, but summaries of the transcripts are available from the corresponding author on reasonable request.
